# Cash Transfers and Their Effect on Maternal and Young Children’s Health: A Randomized Clinical Trial

**DOI:** 10.1001/jamapediatrics.2025.1612

**Published:** 2025-06-16

**Authors:** Greg J. Duncan, Katherine Magnuson, Alicia S. Kunin-Batson, Hirokazu Yoshikawa, Nathan A. Fox, Sarah Halpern-Meekin, Nicholas J. Ainsworth, Sarah R. Black, Jennifer Mize Nelson, Timothy D. Nelson, Michael K. Georgieff, Debra Karhson, Lisa A. Gennetian, Kimberly G. Noble

**Affiliations:** 1 University of California, Irvine; 2 University of Wisconsin-Madison, Madison; 3 University of Minnesota, Minneapolis; 4 New York University, New York; 5 University of Maryland, College Park; 6 Kent State University, Kent, Ohio; 7 University of Nebraska-Lincoln, Lincoln; 8 University of New Orleans, New Orleans, Louisiana; 9 Duke University, Durham, North Carolina; 10 Teachers College, Columbia University, New York, New York

## Abstract

**Importance:**

Mothers and children in low-income households are more likely to experience worse mental and physical health than those from higher-income households.

**Objective:**

To determine the effect of 4 years of monthly unconditional cash transfers on the mental health of mothers with low-income and the physical health of mothers and children.

**Design, Setting, and Participants:**

This was a parallel-group, randomized clinical trial conducted from May 2018 to July 2023. Mother-infant dyads were recruited (May 2018-June 2019) from postpartum wards in 12 hospitals in 4 cities: Omaha, Nebraska; Minneapolis/St Paul, Minnesota; New Orleans, Louisiana; and New York, New York. Data were analyzed from September 2023 to February 2025.

**Interventions:**

Mothers were randomly assigned to receive either a high-cash gift ($333 per month) or a low-cash gift ($20 per month) on debit cards. The cash gifts continued for the first 6 years of their children’s lives. Data analyzed here were collected after 4 years of monthly transfers.

**Main Outcomes and Measures:**

Outcomes were preregistered and measured around the child’s fourth birthday. Maternal outcomes included depression, anxiety, and body mass index (BMI). Child outcomes included age- and sex-adjusted BMI percentile and maternal report of child health (overall health, times sick in the past year, and presence of chronic health conditions).

**Results:**

A total of 1000 mother-infant dyads (mean [SD] maternal age, 27.0 [5.8] years) were included in this study. Among those mothers, 400 were randomly assigned to receive the $333 high-cash gift and 600 received the $20 low-cash gift on debit cards. Data were available from 891 mother-child dyads. No statistically detectable group differences were found in maternal depressive symptoms (effect size [ES], 0.04; 95% CI, −0.08 to 0.17; *P* = .51), anxiety (ES, 0.12; 95% CI, −0.02 to 0.25; *P* = .09), or BMI (ES, −0.06; 95% CI, −0.21 to 0.09; *P* = .42). In addition, there were no statistically detectable group differences in child BMI percentile (ES, −0.03; 95% CI, −0.17 to 0.12; *P* = .73) or overall child health (ES, 0.08; 95% CI, −0.07 to 0.22; *P* = .30).

**Conclusions and Relevance:**

Monthly unconditional cash transfers totaling approximately $15 000 over 4 years to mothers with low incomes did not improve maternal mental health, maternal or child BMI, or maternal report of children’s health. These results could reflect the absence of causal connections between cash transfers and health, the possibility that impacts of early childhood income may not appear until later in life, or that an 18% increase in income is insufficient to overcome the structural vulnerabilities associated with poverty that contribute to health.

**Trial Registration:**

ClinicalTrials.gov Identifier: NCT03593356

## Key Points

**Question:**

Does income support improve the mental health of mothers and the physical health of mothers and their young children?

**Findings:**

In this randomized clinical trial of 1000 mother-child dyads in the US with household income below the poverty line, a monthly unconditional cash transfer of $333 over the first 4 years of life did not improve measures of maternal physical or mental health or the physical health of their 4-year-old children.

**Meaning:**

Results suggest that cash transfers starting at birth over a 4-year period may not be sufficient to address the antecedents of health outcomes in families with low-income.

## Teaser

This randomized clinical trial investigates the effect of income support on both the mental health of mothers and the physical health of mothers and their young children.

## Introduction

Poverty is an important correlate of the health and well-being of US children and their mothers, but little is known about the extent to which cash transfers improve maternal and child health. Children and female individuals, especially Black and Hispanic females, disproportionately experience poverty at high rates,^1^ contributing to health disparities. Low household income has been associated with higher rates of maternal mortality,^2^ as well as obesity and associated cardiometabolic conditions (hypertension, diabetes),^3,4^ and worse maternal mental health.^5^ Compared with those with higher incomes, those experiencing poverty during the early childhood years have been associated with higher obesity and steeper body mass index trajectories,^6,7,8^ worse parent perceptions of children’s overall health,^9^ more frequent illness,^10^ and higher prevalence of chronic conditions.^11,12^ Cash transfers have the potential to alter health and well-being by improving household access to resources (eg, higher-opportunity neighborhoods,^13,14^ health/mental health care,^15^ opportunities for healthy eating^8^ and physical activity^16^) and alleviating the stress of economic hardship.^17^

Although much of the evidence for the role of income on children’s health has come from correlational/observational studies, quasi-experimental studies provide more convincing causal evidence. Evaluations of US policy changes suggest that increasing both the economic resources and employment of families with low income improves health trajectories for children living in low-income households. For example, state-based expansions of the Earned Income Tax Credit (EITC), which is paid only to families with taxable (typically earned) income, have been associated with improved birth outcomes, especially for Black children,^18^ and reductions in behavioral problems and pediatric child abuse.^19,20^ In the case of maternal mental health, national expansions of the generosity of the EITC have been found to improve maternal reports of mental health and an index of biomarkers associated with inflammation and cardiovascular problems.^21^ But because EITC-based policies combine income transfers and employment incentives—and typically lead to greater employment—the health effects of unconditional cash transfers for young children and their mothers in the US are unknown.

The present study evaluated the causal effect of a monthly unconditional cash transfer on child and maternal health after 4 years of receipt. This intervention exemplifies a scalable public health approach. Results from prior analysis of this study showed suggestive positive effects of the intervention on electrophysiologic brain activity at approximately 12 months of age,^22^ but no statistically detectable effects were found on maternal mental health or maternal report of child health outcomes when children were approximately 12, 24, or 36 months of age.^23,24^ Our preregistered hypotheses were that a monthly unconditional cash transfer would lead to improved maternal mental health and maternal assessments of children’s health and reductions in child and maternal BMI.

## Methods

### Study Design Overview

Baby’s First Years (BFY) is a parallel-group, randomized clinical trial of income support to low-income families. Between May 2018 and June 2019, mothers with household incomes below the poverty line were recruited from postpartum wards after giving birth and offered a monthly, unconditional cash transfer (referred to as a *cash gift*). Mothers were randomly assigned to either a high-cash gift group or a low-cash gift group. Monthly gifts were initially promised for the first 40 months of children’s lives. This duration has been extended twice, first to 52 months because of the COVID-19 pandemic’s impact on in-person data collection, and subsequently to 76 months, so as to last for all of early childhood. Outcomes measured at 48 months after randomization (collected between July 2022 and August 2023) are the focus of the current study. Study design details are described in Noble et al^25^ and in Supplement 1 and eAppendixes 1 to 3 in Supplement 2. This study follows the Consolidated Standards of Reporting Trials (CONSORT) reporting guidelines for randomized clinical trials.

On providing written informed consent, mothers completed baseline surveys and received compensation for their participation ($50). For the next 3 years, mothers were invited to complete annual in-person or telephone surveys around the time of their child’s birthday. Around the children’s fourth birthdays, mothers and children were invited to participate in data collection at universities in each of the 4 sites. All study procedures were approved by the institutional review board of Teachers College, Columbia University, under protocol number 18-210.

### Participants

Mothers were recruited from 12 hospitals across 4 US metropolitan areas: New York, New York; Omaha, Nebraska; New Orleans, Louisiana; and Minneapolis/St. Paul, Minnesota. Eligibility criteria included the following: (1) being of legal age to provide consent, (2) reporting a household income below the federal poverty line in the calendar year before the birth, (3) speaking English or Spanish, (4) residing in the state of recruitment, (5) self-reported that a permanent move out of state in the next 12 months was “not at all likely,” (6) singleton infant who had not been admitted to the neonatal intensive care unit, and (7) infant to be discharged into the mother’s custody. Participants selected from the following race and ethnicity categories: American Indian, Eskimo, or Aleut; Asian or Pacific Islander; Black or African American; White; or other. They then self-reported whether they considered themselves Hispanic or Latino or not. Responses to these 2 questions were combined to create the categories used in the analysis: non-Hispanic Black, Hispanic, non-Hispanic multiracial/multiethnic, non-Hispanic White, and unknown or other, which included all other combinations of race and ethnicity.

### Study Procedures

Randomization occurred within each of the 4 cities, with 60% of mothers randomized to the low-cash gift group and 40% randomized to the high-cash gift group (Supplement 1). After obtaining consent, conducting the baseline survey, and obtaining a verbal expression of interest in receiving a monthly cash gift, interviewers retrieved the computer-generated randomized group assignment, informed participants of their assigned group, and activated debit cards with the initial monthly payment. Neither interviewers nor participants were blinded to condition at the time of recruitment.

Mothers in the high-cash gift group received $333 per month ($3996 per year); mothers in the low-cash gift group received $20 per month ($240 per year). The approximately $4000 level of the high-cash gift payments was determined from our literature review of effects on children and families of programs such as the EITC (mentioned previously). It provided (inflation-adjusted) average payments that were smaller than $4000 per family and has been found to improve children’s achievement test scores.

Funds were disbursed monthly onto an electronic debit card (branded *4MyBaby*) and accompanied by a text message notification.^25^ Note that, in contrast to policies such as the Child Tax Credit, the payment amount did not depend on the number of children in the household. The annual receipt of nearly $4000 in cash gifts represented an 18% increase in the baseline average family income of the sample. Ninety-one percent of the participating mothers continued to report family incomes that were below 200% of the federal poverty line across the first 4 years of the study. We worked with state officials to ensure that our cash transfers did not affect eligibility for Medicaid or the eligibility and benefit levels for SNAP (Supplemental Nutrition Assistance Program), childcare assistance, LIHEAP (Low Income Home Energy Assistance Program), and TANF (Temporary Assistance for Needy Families). Mothers were told at the time of recruitment that the cash gifts would count as income for the SSI (Supplemental Security Income) program.

Participants were informed they could spend the money as they wished, and receipt of the funds was not contingent on study participation. Only 5 families had not withdrawn funds from the debit card 3 years after randomization.^26^ No adverse events related to the cash gift treatment were reported, although a programming error by the financial institution administering the debit cards led 24 families to receive (and retain) 1-time overpayments (eAppendix 6 in Supplement 2).

The study was designed so that, after accounting for an expected 20% attrition rate, there would be sufficient statistical power to detect an effect size (ES) of approximately 0.20 SD. An initial sample size of n = 1000 (and expected n = 800 at age 4 years), divided 40%/60% between high- and low-cash gift groups, provided 80% statistical power to detect a 0.20-SD impact at age 4 years with *P* < .05 in a 2-tailed test. Our review of the quasi-experimental literature on income effects in childhood led us to expect that our $313 (= $333 − $20) group monthly payment differential would cause about 0.20-SD differences in the primary child outcomes measured in our study.^27^ Using the Bloom^28^ conversion of SEs into statistical power, subsequent regression results confirmed the accuracy of our original power calculations.

### Measures

Maternal and child health measures were preregistered as secondary outcomes at clinicaltrials.gov. Only the maternal anxiety scale was changed (from the Beck Anxiety Inventory to the Generalized Anxiety Disorder 7 [GAD-7]) after the trial commenced. Measures are briefly described here and detailed in eAppendixes 2, 3, and 4 in Supplement 2. As to maternal mental health, mothers were orally administered the GAD-7^29^ and Patient Health Questionnaire 8 [PHQ-8]^30^ scales. These are brief, widely used screening measures of anxiety and depressive symptoms, with higher scores reflecting greater symptoms. To measure maternal physical health, we measured each mother’s height and weight following standardized protocols during university visits, and calculated body mass index (BMI) by dividing weight in kilograms by height in meters squared.

Children’s height and weight were also measured during university visits and converted to age- and sex-adjusted BMI percentiles using the US Centers for Disease Control and Prevention (CDC) 2000 growth charts.^31^ We preregistered an index of child health indicators with 3 components: overall health, times sick during the last year, and chronic health condition. We called this index the *child overall poor health index* to denote that higher values indicate worse health outcomes. A continuous scale was constructed from these 3 items with index scores ranging from 0 to 8.

### Statistical Analysis

Intention-to-treat analyses of continuous measures were conducted by fitting a linear regression equation with robust SE for each outcome. In the case of dichotomous outcomes reported in the supplemental materials, a logistic regression was fit to the data, and results are reported as marginal changes in the probability of affirmative responses.

Analyses were adjusted for preregistered child and maternal covariates measured at baseline (eAppendix 5 and eTable 1 in Supplement 2), plus the child’s age in months at the time of data collection and indicators for the interviewer who administered the measure. All analyses were conducted from September 2023 to February 2025 using Stata, version 18 (StataCorp).^32^

## Results

### Descriptive Statistics

**Figure 1.**
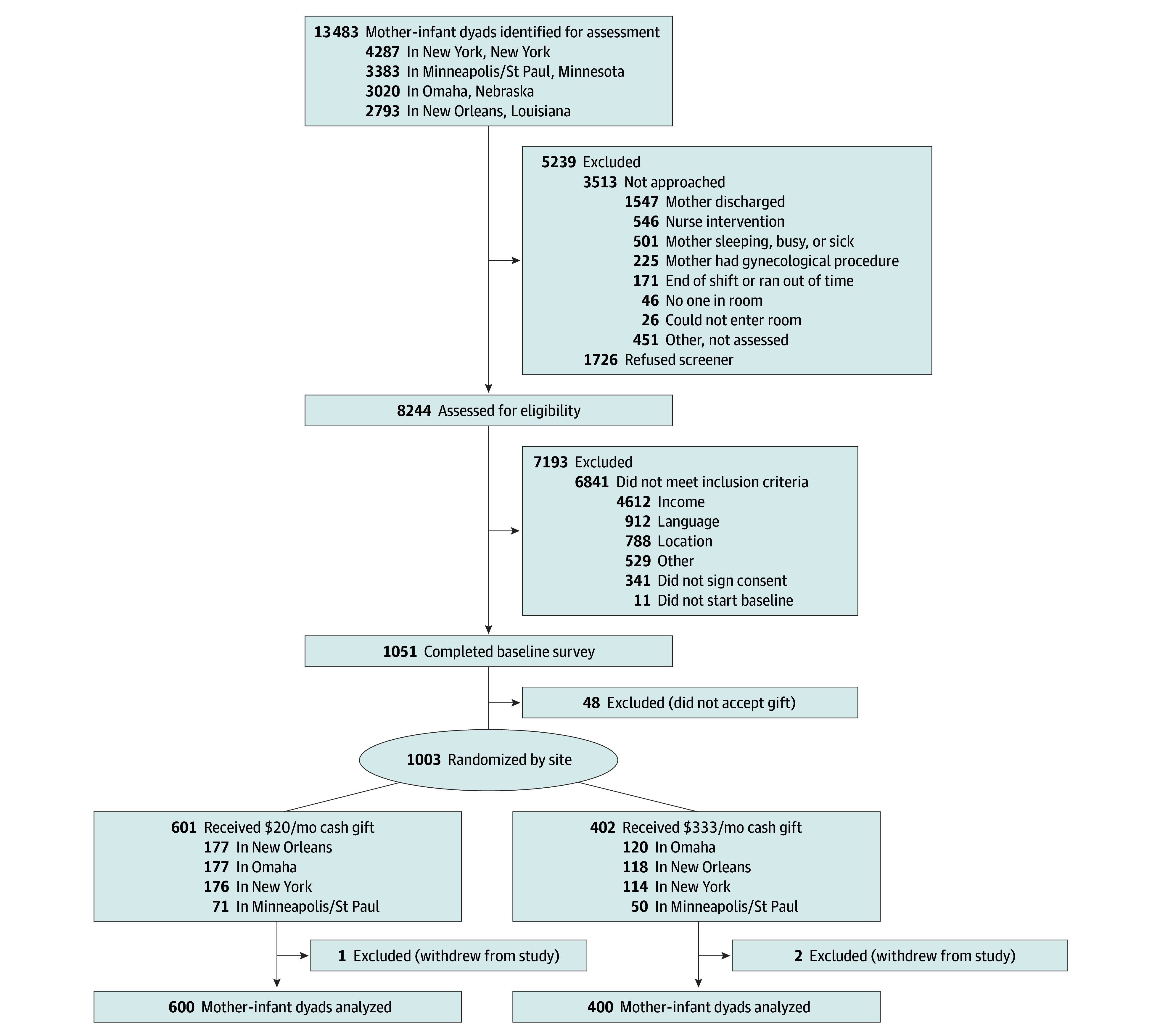
Consolidated Standards of Reporting Trials (CONSORT) Diagram for Recruitment

A total of 1000 mother-infant dyads (mean [SD] maternal age, 27.0 [5.8] years; 414 non-Hispanic Black [41.4%]; 409 Hispanic [40.9%]; 36 non-Hispanic multiracial/multiethnic [3.6%]; 101 non-Hispanic White [10.1%], 40 other or unknown [4.0%]) participated in the study. A total of 400 mothers were randomly assigned to receive the $333 high-cash gift, and 600 received the $20 low-cash gift on debit cards. Table 1 presents baseline descriptive statistics for the overall sample and by treatment status for the analysis sample at age 4 years (n = 891) (Figure 1). On average, mothers had completed close to 12 years of schooling and had between 1 and 2 older children at the time of the birth. A total of 38% of mothers (335 of 891) reported living with the biological father of the baby at the time of the birth. Virtually all infants were of normal birth weight (mean [SD], 3.2 [0.5] kg) and were born at term (mean [SD], 39 [1.2] weeks).

**Table 1.** Sample Characteristics and Baseline Balance for the Low- and High-Cash Gift Groups in the Analytic Sample of Children Aged 4 Years^a^^,^^b^

Characteristic	Overall sample (N = 891)		Low-cash gift group (n = 517)		High-cash gift group (n = 374)		Standardized mean difference^e^	
	Mean (SD)^c^	No.^d^	Mean (SD)	No.	Mean (SD)	No.	Hedges *g*	Cox Index
Child characteristics								
Child is female	0.49	891	0.50	517	0.476	374	NA	−0.06
Child weight at birth, kg	3.23 (0.47)	889	3.24 (0.47)	516	3.22 (0.46)	373	−0.05	NA
Child gestational age, wk	39.07 (1.22)	887	39.11 (1.21)	513	39.017 (1.241)	374	−0.07	NA
Maternal characteristics								
Mother age at birth, y	27.17 (5.88)	891	27.00 (5.93)	517	27.404 (5.822)	374	0.07	NA
Mother education, y	11.90 (2.84)	883	11.91 (2.78)	511	11.882 (2.935)	372	−0.01	NA
Mother race and ethnicity								
Non-Hispanic Black	0.42	891	0.40	517	0.449	374	NA	0.12
Hispanic	0.41	891	0.408	517	0.417	374	NA	0.02
Non-Hispanic multiracial/multiethnic	0.04	891	0.04	517	0.032	374	NA	−0.16
Non-Hispanic White	0.09	891	0.10	517	0.083	374	NA	−0.13
Other or unknown^f^	0.04	891	0.048	517	0.019	374	NA	−0.58
Mother marital status								
Never married	0.45	891	0.420	517	0.497	374	NA	0.19
Single, living with partner	0.24	891	0.253	517	0.211	374	NA	−0.14
Married	0.22	891	0.222	517	0.219	374	NA	−0.01
Divorced/separated	0.04	891	0.046	517	0.029	374	NA	−0.29
Other or unknown	0.05	891	0.058	517	0.043	374	NA	−0.19
Mother health is good or better	0.90	891	0.884	517	0.922	374	NA	0.27
Mother depression (CESD)	0.68	891	0.687	517	0.680	374	−0.01	NA
Cigarettes per week during pregnancy, No.	3.83 (16.07)	884	4.240 (18.741)	512	3.255 (11.426)	372	−0.06	NA
Alcohol drinks per week during pregnancy, No.	0.10 (1.32)	888	0.151 (1.694)	515	0.027 (0.394)	373	−0.09	NA
Children born to mother, No.	2.46 (1.41)	891	2.412 (1.399)	517	2.529 (1.413)	374	NA	0.08
Adults in household, No.	2.06 (0.98)	891	2.083 (0.980)	517	2.027 (0.971)	374	−0.06	NA
Biological father lives in household	0.38	891	0.400	517	0.342	374		−0.15
Household combined income	21 857.49 (19 618.64)	835	22 566.33 (21 824.87)	487	20 865.53 (16 007.76)	348	−0.09	NA
Household income unknown^g^	0.06	891	0.058	517	0.070	374	NA	0.12
Household net worth	−1955.07 (24 329.39)	792	−1802.07 (30 130.01)	458	−2164.86 (12 652.70)	334	−0.02	NA
Household net worth unknown	0.11	891	0.114	517	0.107	374	NA	−0.04

Abbreviations: CESD, Center for Epidemiological Studies–Depression; NA, not applicable.

^a^ Although the total number of respondents providing data for any outcome during the data collection at age 4 years was 891 (Figure 2), the number of respondents providing a response for each health outcome measured in this article ranged from 737 to 884. eAppendix 2 in Supplement 2 provides reasons for outcome nonresponse among the sample of respondents aged 4 years.

^b^ Results from joint tests of the equality of baseline characteristic means for various groups of respondents are provided. Joint tests were conducted using probit models with robust standard errors and site-level fixed effects. Joint tests include all characteristics reported in the table. For continuous variables used in each joint test, the mean for all baseline respondents is imputed for respondents with missing baseline data. The following missing data indicators are also included in each joint test: child weight at birth, mother’s education, number of cigarettes per week during pregnancy, and number of alcohol drinks per week during pregnancy. For all samples, missing baseline data on child gestational age perfectly predicted failure in the probit regression models and is dropped from the joint tests. Additionally, the number of cigarettes per week during pregnancy perfectly predicted failure in the probit regression model for the sample of respondents to child’s BMI percentile and is dropped from the joint test for that outcome only (eTable 1 in Supplement 2). Joint test of the equality of means across all baseline characteristics for participants providing a response to at least one outcome: χ^2^_28_ = 30.91; *P* = .32; n = 891. Joint tests of the equality of means across all baseline characteristics for participants providing a response for each outcome: GAD-7 total score: χ^2^_28_ = 28.64; *P* = .43; n = 883; PHQ-8 total score: χ^2^_28_ = 28.64; *P* = .43; n = 883; mother’s BMI: χ^2^_28_ = 25.03; *P* = .63; n = 737; child overall poor health index: χ^2^_28_ = 31.16; *P* = .31; n = 884; child’s BMI percentile: χ^2^_27_ = 26.87; *P* = .47; n = 811.

^c^ The mean of each child and maternal characteristic at baseline for participants that provided data for at least 1 outcome are reported for the overall sample and separately for the low-cash and high-cash gift groups, with SDs reported in parentheses for continuous variables only.

^d^ Sample sizes for respondents with nonmissing data for each characteristic at baseline are reported for the full sample and separately for the low-cash and high-cash gift groups.

^e^ Standardized mean differences between the low-cash and high-cash gift groups were calculated using Hedges *g* for continuous variables and Cox Index for dichotomous variables.

^f^ Other race and ethnicity includes all other combinations of race and ethnicity.

^g^ If there were more than 10 missing cases for a baseline characteristic, missing data indicators were included in the table and the joint tests. If there were less than 10 cases missing, missing data indicators were not included in the table but were included in the joint tests.

**Figure 2.**
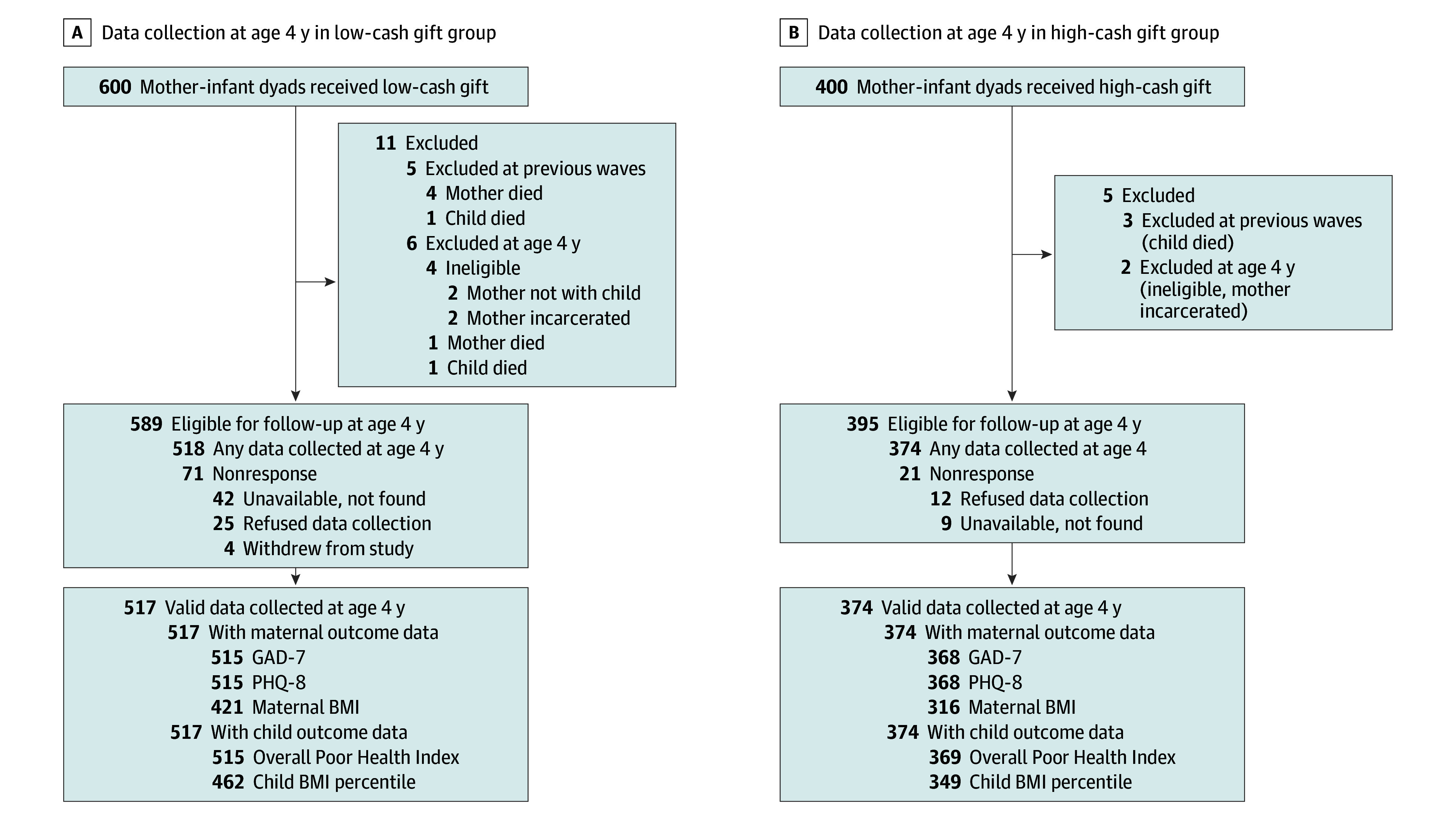
Consolidated Standards of Reporting Trials (CONSORT) Diagram for Data Collection at Age 4 Years BMI indicates body mass index; GAD-7, Generalized Anxiety Disorder 7; PHQ-8, Patient Health Questionnaire 8.

At the beginning of data collection at age 4 years, 984 dyads were eligible for study participation owing to 5 maternal deaths, 5 child deaths, 2 maternal-child separations, and 4 instances of maternal incarceration (Figure 2). As explained subsequently, the number of available observations for the 5 preregistered assessments of maternal and child health ranged from 737 to 884. The fraction of our eligible high-cash and low-cash gift groups providing at least some data on outcomes was 94.7% (374 of 395) and 87.8% (517 of 589), respectively. Differential response rates favoring the high-cash gift group characterized all 5 of the outcomes included in our impact analyses. eAppendix 5 in Supplement 2 estimates the sensitivity of the results to various adjustments for differential nonresponse.

Descriptive statistics for primary outcomes are presented in Table 2^33,34^ and eTable 2 in Supplement 2. For the full sample, the mean (SD) maternal GAD-7 score was 4.80 (4.96) and PHQ-8 score was 4.32 (4.81). Rates of clinically significant maternal anxiety (17.4% [154 of 883]) and depression (14.3% [126 of 883]) symptoms were similar to rates observed among mothers with low income in a large national sample during the same time period.^35^ Mean (SD) of BMI for mothers was 31.05 (8.18). The mean of children’s BMI was at the 64th percentile (mean [SD], 64.30 [30.96]). Among participating mothers and children, rates of obesity were somewhat high relative to population estimates (ie, 378 of 737 mothers [51.3%] experienced obesity, compared with 42%, on average, for adult women; 156 of 811 children [19.2%] experienced obesity, compared with 13%, on average, for children aged 2-5 years).^36,37^ Incidence of childhood chronic conditions was low, consistent with the young age of the cohort. The most common condition was asthma, which was reported by 31 of 884 mothers [3.5%] in the sample. The mean (SD) for the continuous scale of the child overall poor health index (overall health, times sick during the last year, and chronic health condition) was 1.75 (1.46).

**Table 2.** Descriptive Statistics and Cash Gift Treatment Effects on Preregistered Maternal and Child Health Outcomes at Age 4 Years^a^

Outcome	Overall mean (SD)^b^	Low-cash gift group mean (SD)	High-cash gift group mean (SD)	Regression-adjusted difference in means (95% CI)^c^	Standardized effect size (95% CI)^d^	*P* value	Westfall- Young adjusted *P* value^e^	No.
Preregistered maternal health outcomes^f^								
GAD-7 total score	4.80 (4.96)	4.59 (4.91)	5.10 (5.02)	0.57 (−0.10 to 1.24)	0.12 (−0.02 to 0.25)	.09	.15	883
PHQ-8 total score	4.32 (4.81)	4.21 (4.95)	4.46 (4.61)	0.21 (−0.42 to 0.84)	0.04 (−0.08 to 0.17)	.51	.50	883
Mother’s BMI	31.05 (8.18)	31.28 (8.43)	30.75 (7.83)	−0.50 (−1.73 to 0.73)	−0.06 (−0.21 to 0.09)	.42	NA	737
Preregistered child health outcomes^f^								
Child overall poor health index	1.75 (1.46)	1.71 (1.42)	1.79 (1.50)	0.11 (−0.09 to 0.31)	0.08 (−0.07 to 0.22)	.30	NA	884
Child’s BMI percentile	64.30 (30.96)	64.64 (30.52)	63.85 (31.58)	−0.80 (−5.30 to 3.71)	−0.03 (−0.17 to 0.12)	.73	NA	811

Abbreviations: BMI, body mass index; NA, not applicable.

^a^ Detailed information describing each outcome measure and its construction can be found in eAppendix 2 in Supplement 2.

^b^ The mean outcome of respondents is reported overall and separately for the low-cash and high-cash gift groups with SDs.

^c^ Results in the fourth column are adjusted for both site fixed effects and preregistered control measures. Covariates include the following maternal self-report covariates from the Baby’s First Years baseline survey conducted at the time of enrollment: mother’s age, completed maternal schooling, household income, net worth, general maternal health, maternal mental health, maternal race and ethnicity, marital status, number of adults in the household, number of other children born to the mother, maternal smoking during pregnancy, maternal alcohol consumption during pregnancy, father living with the mother, child’s sex, child’s birth weight, and child’s gestational age at birth. Models also control for the child’s age (in months) at the time the measure was collected and for interviewer ID. Missing data for covariates impute the mean value from the given analytic sample. All models include indicators for missing data on baseline covariates when missing data are present. Descriptions of each covariate used in the model can be found in eTable 1 in Supplement 2.

^d^ Numbers convert fourth column differences into effect sizes with division by low-cash gift group SDs. All 95% CIs are calculated using robust SEs obtained from regression estimates.

^e^ *P* values, both unadjusted and then adjusted for multiple hypothesis testing, are reported. Multiple hypothesis adjustments are made using the step-down resampling methodology of Westfall and Young^33^ to control for the family-wise error rate. To implement this approach, we used the Stata package (StataCorp) developed by Jones et al.^34^ Maternal mental health measures (GAD-7 and PHQ-8) were preregistered as belonging to one family of outcomes for the purposes of multiple hypothesis testing. Each of the other measures (mother’s BMI, child’s BMI percentile, and child overall poor health index) was preregistered as belonging to its own family. Therefore, we omit *P* values adjusted for multiple hypothesis testing for these outcomes only, which are denoted with NA.

^f^ All maternal and child health measures were preregistered as secondary outcomes.

### Impacts on Maternal Outcomes

Regression results (Table 2)^33,34^ show that the cash gift amount produced no statistically significant effects on the preregistered maternal health outcomes. There were no statistically significant group differences in maternal GAD-7 scores (ES, 0.12; 95% CI, −0.02 to 0.25; *P* = .09), PHQ-8 scores (ES, 0.04; 95% CI, −0.08 to 0.17; *P* = .51), or BMI (ES, −0.06; 95% CI, −0.21 to 0.09; *P* = .42). In exploratory analyses (eAppendix 4 in Supplement 2), there were no significant differences in rates of clinically meaningful depression symptoms (indicated by moderately severe to severe elevations) or anxiety symptoms (scores at or above the moderate range) by group. In 1 case—meeting at least the mild symptom threshold on the PHQ-8—the estimated coefficient was significant but its positive direction was contrary to expectations.

### Effects on Child Health Outcomes

There were no statistically significant group differences in child BMI percentile (ES, −0.03; 95% CI, −0.17 to 0.12; *P* = .73) or overall child health (ES, 0.08; 95% CI, −0.07 to 0.22; *P* = .30) between the high-cash and low-cash gift groups. In exploratory analysis (eTable 2 in Supplement 2), we modeled each component of the poor health index separately and did the same for child BMI percentile thresholds, finding no significant group differences in any of these outcomes.

Finally, we investigated whether the results were sensitive to weighting-based approaches to baseline imbalance, nonresponse bias, multiple imputation, and overpayment, and we failed to find evidence for this (eAppendixes 5 and 6 and eTables 3 and 4 in Supplement 2).

## Discussion

This preregistered study found that 4 years of monthly unconditional cash transfers for families with low household incomes did not improve measures of either maternal mental health or maternal reports of child health. Additionally, there were no treatment differences in BMI for either mothers or children. These patterns of null effects contrast with expectations based on correlational studies but are consistent with findings at earlier waves of BFY trial data collection, which found no statistically detectable effects on mothers’ reports of their own mental health, child health, or medical care.^23,24^

Our failure to reject the null effects hypotheses in the domain of maternal mental health may be due to the lack of impact of cash gifts on parents’ stress, as higher cash gifts did not improve maternal reports of economic or material hardship nor perceived stress during the first 3 years of children’s lives.^24^ Because these are key mechanisms that might lead to worsened mental health, it is perhaps unsurprising that we saw no differences in mothers’ symptoms of depression and anxiety.

Past BFY trial work found that cash gifts did not lead to better housing quality or increase moving to better-resourced neighborhoods,^38^ both of which might have lowered rates of asthma,^39^ obesity,^40^ or other chronic illness.^41^ Changes in children’s dietary intake among the high-cash gift group—specifically an increase in fruit and vegetable consumption as reported at age 2 years—were not accompanied by other dietary changes (eg, reduction in sweets/sugared beverages)^23^ that would likely be necessary to yield reductions in child BMI. Additionally, we previously reported no early effects on child sleep or health care utilization, including both preventive and emergent health care services, which could also be expected to influence health outcomes in children.^23^

An alternative explanation for lack of significant group differences centers on the turbulent conditions associated with the COVID-19 pandemic, which coincided roughly with the time between the first and third birthdays of children in the BFY trial. The public health and social effects of the COVID-19 pandemic may have harmed family well-being by increasing caregiving demands, isolation, and other challenges that are unlikely to be buffered by cash gifts. Surprisingly, though, the COVID-19 pandemic did not appear to cause major disruptions to family well-being in this cohort.^42,43^

The economic context of families also fluctuated during the COVID-19 pandemic, with 60% of mothers in the low-cash gift group and 67% of mothers in the high-cash gift group reporting some lost household income during this time. At the same time, most also reported receiving enhanced government support, particularly from the economic relief legislation passed in 2021, including tax credits, expansions in unemployment insurance and food assistance, and eviction moratoria. Indeed, according to a tax simulation program, BFY trial mothers were eligible in 2021 for approximately $15 000 from expanded COVID-19 pandemic–related government benefits—an amount that was approximately double what they received from these sources in 2020.^44^ These dramatic changes may have reduced overall stress and anxiety during this period. However, cash-gift effects on the time and money parents spent on their child did not fluctuate over this period,^26^ nor did the cash gift effects on maternal-reported child health and developmental outcomes.^23^

Finally, it is possible that unconditional cash transfers do not causally affect the health of mothers and their young children. Households experiencing poverty simultaneously experience multiple stressors, negative life events, and systemic and structural disadvantage that cumulatively contribute to mental and physical health outcomes.^45,46,47,48^ Although household income supplementation would be expected to improve the environmental circumstances of families, our findings suggest that this amount of income alone may have been insufficient to address the specific environmental antecedents of the various maternal and child health outcomes measured here.

More definitive conclusions may be drawn from future waves of data collection. As healthy newborns tend to grow into healthy preschoolers, the effects of early childhood poverty reduction on children’s health may not be fully felt until later in life. As the BFY trial cash transfers are ongoing, it will be possible to determine whether 2 more years of cash transfers alter health outcomes at age 6 years. Longer-term follow-up would enable measuring effects on health conditions such as hypertension and type 2 diabetes that emerge over time among mothers and children.

### Strengths and Limitations

Strengths of this study include its experimental design and low attrition, which represent improvements over past observational studies. Limitations include a narrow range of health outcomes assessed in-person (ie, BMI only) and reliance on parental reports of children’s health.

## Conclusions

In this randomized clinical trial, results reveal that monthly unconditional cash transfers to mothers with incomes below the poverty line, totaling approximately $15 000 or an 18% increase in income over the first 4 years of their child’s life, produced no improvements in maternal mental health, parent-reported child health, or BMI of mother or child. The lack of health-related differences may reflect the absence of causal connections between household income and health or may indicate that these cash transfers alone were insufficient to alleviate the complex structural and systemic disadvantages that contribute to both mental and physical health. As the effects of early childhood income support on health outcomes may not emerge until later in development or much later in the life course, continued follow-up will be important to better understand the effects of unconditional cash transfers for maternal and child health.

## Notes

Supplement 1.
Trial Protocol.

Supplement 2. **eAppendix 1.** Randomization Procedures **eAppendix 2.** Health Measures **eAppendix 3.** Baseline Control Measures **eAppendix 4.** Impacts on Alternative Maternal and Child Health Measures **eAppendix 5.** Impacts Using Various Nonresponse Adjustments **eAppendix 6.** Cash Gift Overpayments
eReferences.
**eTable 1.** List of Baseline Covariates Included in the Impact Regressions **eTable 2.** Descriptive Statistics and Cash Gift Impacts on Exploratory Maternal and Child Health Outcomes at Age 4 **eTable 3.** Cash Gift Impacts on Preregistered Maternal and Child Health Outcomes at Age 4 With Adjustments for Nonresponse **eTable 4.** Sensitivity of Cash Gift Impacts on Maternal and Child Health Outcomes at Age 4 to Overpayments

Supplement 3.
Data Sharing Statement.
