# Supplementary material for: Cash Transfers and Their Effect on Maternal and Young Children’s Health: A Randomized Clinical Trial
Source: JAMA Pediatr. 2025 Jun 16;179(8):867–75. doi: 10.1001/jamapediatrics.2025.1612 (PMC12171960; doi:10.1001/jamapediatrics.2025.1612)
Supplement: Supplement 2. — eAppendix 1. Randomization Procedures eAppendix 2. Health Measures eAppendix 3. Baseline Control Measures eAppendix 4. Impacts on Alternative Maternal and Child Health Measures eAppendix 5. Impacts Using Various Nonresponse Adjustments eAppendix 6. Cash Gift Overpayments eReferences. eTable 1. List of Baseline Covariates Included in the Impact Regressions eTable 2. Descriptive Statistics and Cash Gift Impacts on Exploratory Maternal and Child Health Outcomes at Age 4 eTable 3. Cash Gift Impacts on Preregistered Maternal and Child Health Outcomes at Age 4 With Adjustments for Nonresponse eTable 4. Sensitivity of Cash Gift Impacts on Maternal and Child Health Outcomes at Age 4 to Overpayments [file jamapediatr-e251612-s002.pdf]

Supplementary Online Content

Duncan GJ, Magnuson K, Kunin-Batson AS, et al. Cash transfers and their effect on maternal and young children's health: a randomized clinical trial. *JAMA Pediatr*. Published online June 16, 2025. doi:10.1001/jamapediatrics.2025.1612

eAppendix 1. Randomization Procedures

eAppendix 2. Health Measures

eAppendix 3. Baseline Control Measures

eAppendix 4. Impacts on Alternative Maternal and Child Health Measures

eAppendix 5. Impacts Using Various Nonresponse Adjustments

eAppendix 6. Cash Gift Overpayments

eReferences.

eTable 1. List of Baseline Covariates Included in the Impact Regressions

eTable 2. Descriptive Statistics and Cash Gift Impacts on Exploratory Maternal and Child Health Outcomes at Age 4

eTable 3. Cash Gift Impacts on Preregistered Maternal and Child Health Outcomes at Age 4 With Adjustments for Nonresponse

eTable 4. Sensitivity of Cash Gift Impacts on Maternal and Child Health Outcomes at Age 4 to Overpayments

This supplementary material has been provided by the authors to give readers additional information about their work.

© 2025 Duncan GJ et al. *JAMA Pediatr*.

eAppendix 1: Randomization procedures

Randomization occurred within each of the four sites. The first step in the randomization process was to create four rosters of 250 rows each, with 150 rows designated as "low-cash gifts" and 100 designated as "high-cash gifts". Each of the four 250-row rosters was then randomly ordered. Rows were assigned consecutively-numbered cash gift IDs. The resulting roster data on high vs. low RCT cash gift condition and cash gift IDs were then stored on the survey contractor's server.

As the May, 2018 to June, 2019 recruitment period in hospitals during the immediate postnatal period proceeded, it became clear that IRB and other institutional issues in one site (the Twin Cities) would lead to fewer than 250 recruited participants. This led to a roughly equal increase in the number of roster rows in the other three sites. To accomplish this, additional roster rows were created in each of these sites using the same randomization procedure. When aggregated, the 1,000-row roster matched exactly the 40%/60% distribution of cash gifts across all possible respondents.

The second step was to create a web-based application that, when interfacing with the interviewers' questionnaire software program, could access these rosters, determine the high- vs. low-cash gift condition to be offered to each participant, record that the condition was offered, and return the gift value for the interviewer to share with the participant.

The interviews themselves were conducted using the Blaise-based Computer-Assisted Personal Interview program (version 4.8). After a participant was successfully recruited in the hospital and agreed to receive a cash gift, the Blaise instrument accessed the web application with a pre-loaded link. The web-based application collected information on the site and the IDs of both the respondent and interviewer from the Blaise software for validation purposes. Once that information was processed, the web application accessed the randomized roster for the specified site, retrieved the next available cash gift ID and its amount, and recorded for which respondent, on which date, and by which interviewer it has been claimed. These requests for cash gift assignments could have come from any of the hospitals within the site and were processed in the order in which they were received by the web-based application. Cash gift amounts, the cash gift ID and respondent ID were displayed (via Blaise) to the interviewer for confirmation, along with the randomly-generated monthly gift amount. The interviewer confirmed the information and proceeded with the interview by announcing the cash gift amount to the respondent and setting up a debit card with that amount so that the participant could use it right away.

Taken together, these procedures ensured a randomization process in which the interviewers could not influence the assigned amount. At the same time, the procedures unblinded both the participant and the interviewer to the gift amount during the recruitment period. In subsequent rounds of data collection, interviewers were not reminded (or, in the case of different interviewers, informed) of participants' treatment status during follow-up assessments. Of course, participants were reminded of the cash gift amount on a monthly basis.

eAppendix 2: Health measures

Outcome data were gathered in university visits and, when necessary, by telephone with the mother when children were 4 years of age. The BFY study follows the consolidated standards of reporting trials (CONSORT) guidelines for RCTs. As shown in the CONSORT diagram in Figure 2, at the beginning of the age-4 data collection, the n=1,000 potential participants had fallen to 984 owing to five maternal deaths, five child deaths, two maternal-child separations, and four instances of maternal incarceration. The total number of participating dyads contributing any maternal or child-based health-related data is 891. As explained below, the number of available observations for our five pre-registered assessments ranges from n=737 to n=884. Impacts when participant data are weighted to adjust for differential nonresponse are provided in eAppendix 5.

All of the health-related measures included in the analysis were preregistered as secondary outcomes at clinicaltrials.gov (ID: NCT03593356). As reported in eAppendix 3, in exploratory analyses, we also estimate impacts on several outcomes that were not preregistered. These include the individual components of the child health index (described below), Body mass index (BMI) cutoffs for underweight, overweight, and obesity for mothers and children, as well as cutoffs for various clinical thresholds of the PHQ-8 (depressive symptoms) and GAD-7 (anxiety) for mothers.

Maternal Health Outcomes

Maternal depression and anxiety. Anxiety was measured by the Generalized Anxiety Disorder-7 scale (GAD-7) [1], which is a 7-item additive index with scores ranging from 0 to 21 (Mean=4.80, SD=4.96). (Our original preregistration specified that anxiety would be assess with the Beck Anxiety Inventory. Preregistrations for age 2 and

© 2025 Duncan GJ et al. *JAMA Pediatr*.

beyond changed the measures to the GAD-7 owing to insufficient variation with the Beck.) For each item, mothers were asked how many days in the past two weeks they were bothered by seven different feelings or experiences with response options as follows: “not at all” (0), “several days” (1), “more than half the days” (2), and “nearly every day” (3). For estimating cash gift impacts, we use the GAD-7 scale as a continuous measure. Among the BFY sample, the GAD-7 has shown high levels of internal consistency (α = .90-.92) across the three waves in which it was asked. Across these three waves, 7-17% of mothers were above the suggested clinical cutoff indicating moderate or higher levels of anxiety.

Depression was measured by the Personal Health Questionnaire Depression Scale (PHQ-8)[2], which is an 8-item additive index with scores ranging from 0 to 24 (Mean=4.32, SD=4.81). Similar to the GAD-7, mothers were asked about the number of days within the past two weeks that they were bothered by feelings or experiences. Response options are the same as those for the GAD-7. For estimating cash gift impacts, we use the PHQ-8 scale as a continuous measure. The PHQ-8 has shown high levels of internal consistency across waves (α=.84). Across all four waves of data collection, 8-14% of mothers scored above the suggested clinical cutoff indicating moderate or higher levels of depression.

Valid responses for the GAD-7 and PHQ-8 were collected for 883 mothers. Reasons for non-response on both measures were as follows: 1 refused to answer any survey items; 2 did not complete a follow-up phone survey after completing an in-person lab visit; and 5 declined to answer some survey questions.

Maternal body mass index. Maternal height was measured to the nearest centimeter using a free-standing stadiometer, and weight measured on identical scales (Health O Meter Professional) at all four data collection sites. Body mass index (BMI) was calculated as [weight (kg) / [height (m)]^2] (Mean=31.05, SD=8.18). We estimate impacts on BMI as a continuous measure and, in supplemental analyses, estimate impacts on meeting underweight (defined as BMI < 18.5), overweight (defined as BMI ≥ 25 and < 30) and obesity (defined as BMI ≥ 30) thresholds in accordance with definitions from Centers for Disease Control and Prevention (CDC). Valid maternal BMI data were collected for 737 respondents. Among the sample of mothers providing any data for age 4 health outcomes (n=891), reasons for maternal BMI non-response were as follows: 39 did not complete an in-person lab visit; 17 completed a lab visit in a remote site; 67 were pregnant; 7 did not consent for BMI collection; 3 experienced errors in data collection or recording; and 21 had other reasons such as not having enough time or possible refusal.

Child Health Outcomes

Child body mass index percentile. Child height and weight measurement also followed standardized protocols using free-standing stadiometers and identical Health O Meter Professional scales at all four data collection sites. Body mass index was calculated using the same formula used for mothers’ BMI. Aged-based percentiles by sex were then assigned according to CDC definitions (Mean=64.30, SD=30.96).[3] We estimate cash gift impacts on BMI percentile as a continuous measure and additionally estimate impacts on meeting the underweight (defined as BMI < 5th percentile), overweight (defined as BMI ≥ 85th and < 95th percentile) and obesity (defined as BMI ≥ 95th percentile) thresholds for children in accordance with CDC definitions. Valid child BMI data were collected for 811 respondents. Reasons for outcome nonresponse on child BMI were as follows: 39 did not complete an in-person lab visit; 16 completed a lab visit in a remote site; 4 did not consent for BMI collection; 2 experienced errors in data collection or recording; 8 were not present at the lab visit with their mother; and 11 had other reasons such as not having enough time or possible refusal.

Child overall poor health index. We operationalized child health using an index of maternal-reported child health created by the study team. This *child overall poor health index* has three items, including maternal report of child’s overall health (ranging from 0 “excellent” to 4 “poor”), the number of days the child was sick in the past year (0 “0-1”, 1 “2-3”, 2 “4-6”, and 3 “7 or more”) and whether the child has a chronic health condition (1 if yes, 0 otherwise). A continuous scale was constructed from these three items with index scores ranging from 0 to 8 (Mean=1.75, SD=1.46). Valid responses to the child overall poor health index were collected for 884 respondents. Reasons for outcome nonresponse on this measure were as follows: 1 refused to answer any survey items; 2 did not complete a follow-up phone survey after completing an in-person lab visit; and 4 declined to answer some survey questions.

Child overall poor health was assessed from maternal responses to the question of how they would describe their child’s health, with response categories of excellent, very good, good, fair, or poor. No recall period was included in the question prompt. A continuous scale was constructed and reverse coded, with values ranging from 0 “excellent” to 4 “poor” (Mean=0.70, SD=0.90).

© 2025 Duncan GJ et al. *JAMA Pediatr*.

Frequency of child sickness was measured from maternal responses to the question of how many times in the last year the child was sick, with the following response categories given: 0-1 times, 2-3 times, 4-6 times, and 7 or more times. A continuous scale ranging from 0 to 3 was constructed from these responses by assigning the following values to the number of sick days: 0 “0-1”, 1 “2-3”, 2 “4-6”, and 3 “7 or more” (Mean=0.97, SD=0.84).

Child chronic health condition was assessed by maternal responses to whether the child has been diagnosed with any chronic health condition. Affirmative responses are scored as one and negative responses are scored as zero (Mean=.08). Mothers who endorsed “yes” on the item were asked to specify the diagnosis and these responses were coded. eTable 2 reports cash gift impacts on the most frequently endorsed condition, which was asthma.

eAppendix 3: Baseline control measures

Details on the construction of baseline control measures are presented in eTable 1.

eAppendix 4: Impacts on alternative maternal and child health measures

In addition to the preregistered analyses shown in Table 2, we also estimate impacts of the cash gift on a set of exploratory health outcomes and present them in eTable 2. In Panel A, we estimate the impact of the cash gift on a set of dichotomous outcome variables using logistic regressions and report the average marginal effect, or average marginal change in the predicted probability of an affirmative response. As with the primary specifications for the preregistered analyses, we include site fixed effects and a set of baseline covariates to account for within site randomization and to improve the precision of the estimates. However, because some of the outcomes are low probability events, certain covariates perfectly predicted failure in the logistic regression models. In order to estimate logistic regression models, we identify the covariates that perfectly predict failure for each outcome and either combine them with other covariates (for example, collapsing levels of education to fewer categories) or drop those covariates from the model. The exact lists of covariates used for each model are available upon request.

For maternal mental health as well as maternal and child BMI, we estimate cash gift impacts on meeting clinically relevant thresholds. Thresholds for meeting mild, moderate, and severe generalized anxiety disorder symptoms on the GAD-7 were taken from Spitzer et al. [1] and are defined as scores higher than 5, 10, and 15, respectively. Thresholds for meeting mild, moderate, moderately severe, and severe depressive symptoms on the PHQ-8 were taken from Kroenke et al. [2] and are defined as total scores higher than 5, 10, 15, and 20, respectively. Thresholds for meeting the classification of underweight, overweight, or obese were taken from the CDC. For adults, underweight is defined as a BMI less than 18.5, overweight is defined as a BMI greater than or equal to 25 and less than 30, obese is defined as a BMI greater than or equal to 30 and overweight or obese is defined as a BMI greater than or equal to 25. For children, underweight is defined as a BMI less than the 5th percentile, overweight is defined as a BMI greater than or equal to the 85th percentile and less than the 95th percentile, obese is defined as a BMI greater than or equal to the 95th percentile and overweight or obese is defined as a BMI greater than or equal to the 85th percentile. Children’s chronic health conditions are not based on clinical assessments but rather maternal survey reports of any chronic health condition or asthma.

In Panel B, we present exploratory impacts of the cash gift on continuous outcome variables. These variables comprise two of the three measures included in the pre-registered index of child overall poor health (the third is child chronic health diagnosis, which is included in Panel A). Ordinary Least Squares (OLS) regression models with site fixed effects and covariates are used to estimate cash gift impacts on each standardized outcome.

Overall, we find little evidence that the high-cash gift had impacts on these exploratory maternal and child health outcomes. The only statistically significant impact was on the PHQ-8. Specifically, the high-cash gift led to a statistically significant increase in the probability of meeting the mild threshold or higher for depressive symptoms (6.9 percentage points, $P<.05$), which is in the opposite direction of the hypothesized effect. We do not find evidence that the cash gift reduces the probability that mothers or children are overweight or obese nor does the cash gift reduce the likelihood of mothers reporting that their child has any type of chronic health condition.

eAppendix 5: Impacts using various nonresponse adjustments

To assess the sensitivity of our estimates to sample attrition at age 4, we constructed two types of nonresponse weights using the Toolkit for Weighting and Analysis of Nonequivalent Groups (TWANG) package developed by researchers at the Rand Corporation. [4] TWANG uses generalized boosted regression modeling to estimate propensity scores based on a set of baseline pre-treatment characteristics and generates the specified analytic weights using these estimated propensity scores. We produce estimates using two different nonresponse weighting approaches.

© 2025 Duncan GJ et al. *JAMA Pediatr*.

The first set were inverse probability of treatment weights (IPTW). To construct these weights, the low-cash gift group respondents at age 4 were weighted by the inverse probability of being a high-cash gift group respondent. Although treatment assignment is random and low-cash gift group respondents have 0 probability of being a high-cash gift respondent post random assignment, in practice, low-cash gift group respondents are weighted by the probability of looking similar to the high-cash gift group respondents that participated in age 4 data collection (i.e., those who did not attrit). What these weights accomplish is balancing the baseline characteristics of low-cash gift group respondents to the characteristics of high-cash gift group respondents in our sample at age 4. The resulting estimand can be characterized as an "average treatment effect on the treated" or ATT. To construct these IPTW, 3 separate weights were constructed for responding to the survey measures (maternal mental health outcomes and the child health index), participating in the child BMI data collection, and participating in the maternal BMI data collection.

We also constructed nonresponse weights (NRW). To construct them, all respondents were weighted by the inverse probability of being an age 4 respondent based on characteristics observed at baseline. These weights attempt to adjust for sample attrition by balancing the baseline characteristics of respondents to the characteristics of the full BFY sample at baseline. As such, the resulting estimand can be characterized as an "average treatment effect" or ATE. To construct the NRW, 3 separate weights were constructed for responding to the survey measures (maternal mental health outcomes and the child health index), participating in the child BMI data collection, and participating in the maternal BMI data collection.

Impact estimates using these different weighting approaches are presented in eTable 3. We also present the unweighted impact estimates and *P* values for comparison. Across outcomes, the weighted impact estimates are very similar to the unweighted estimates from our preregistered model specifications. Moreover, there are no instances in which weighting leads to conclusions about statistical significance that differ from our main impact estimates.

We next estimated the impact of the BFY high-cash gift on preregistered outcomes using multiple imputation to account for missing data. We imputed 20 datasets with multiple imputation by chained equations, or MICE, using predictive mean matching. As shown in eTable 3, multiple imputation produces a pattern of results similar to those shown in Table 2. Overall, adjustments for sample attrition do not change our overall conclusions that the high-cash gift did not affect maternal and child health outcomes.

eAppendix 6: Cash gift overpayments

On two occasions in the four-year treatment period (when children were ages 2 and 4), the financial institution distributing BFY's monthly cash gifts mistakenly provided over-payments to 24 families. Eight of the families were in the low-cash gift group and 16 were in the high-cash gift group. The mean [SD] overpayment for those receiving one was $1,293 [$1,234]. Corresponding means and standard deviations for the low- and high-cash gift groups were $93 [$49] and $1,894 [$1,086]. For the age 4 data collection, 22 families that ever received an overpayment of the cash gift provided data for at least one maternal or child health outcome, including 14 from the high-cash gift group and 8 from the low-cash gift group.

We investigated the possible impact of overpayments on our treatment results in two ways (eTable 4). First, we added a dichotomous control variable to the impact regressions shown in Table 2. The resulting effect sizes and significance levels (top panel of eTable 4) are virtually identical to their Table 2 counterparts. Second, we estimated regressions leaving out the 22 cases with overpayments. Here again, the resulting effect sizes and significance levels (bottom panel of eTable 4) are virtually identical to their Table 2 counterparts.

eReferences

1. Spitzer RL, Kroenke K, Williams JW, Löwe B. A brief measure for assessing generalized anxiety disorder: The gad-7. *Archives of Internal Medicine*. 2006;166(10):1092-1097. doi:10.1001/archinte.166.10.1092
2. Kroenke K, Strine TW, Spitzer RL, Williams JB, Berry JT, Mokdad AH. The PHQ-8 as a measure of current depression in the general population. *Journal of affective disorders*. 2009;114(1-3):163-173.
3. Kuczmarski RJ. *CDC growth charts: United States*. US Department of Health and Human Services, Centers for Disease Control and …; 2000.
4. Griffin BA, Ridgeway, G., Morral, A.R., Burgette, L.F., Martin, C., Almirall, D., Ramchand, R., Jaycox, L.H., McCaffrey, D.F. Toolkit for Weighting and Analysis of Nonequivalent Groups (TWANG) Website Santa Monica, CA: RAND. http://www.rand.org/statistics/twang

© 2025 Duncan GJ et al. *JAMA Pediatr*.

eTable 1. List of Baseline Covariates Included in the Impact Regressions[a]

Measure	Explanation
Child is female	Dichotomous, based on maternal report at baseline
Child weight at birth	Birthweight in pounds, based on maternal report of birthweight in pounds and ounces at baseline
Child gestational age	Child's gestational age at birth in weeks, based on a comparison of due date from maternal report at baseline and date of birth as recorded in hospital records and verbally confirmed by mother in the baseline interview
Mother age at birth	Mother age in years at time of child's birth, based on maternal report at baseline
Mother education	Coded into six mutually exclusive indicators (less than high school degree; high school degree; some college; associate's degree; bachelor's degree or more; unknown), based on maternal report at baseline
Mother race/ethnicity	Coded into six mutually exclusive indicators (Non-Hispanic, White; Non-Hispanic, Black or African American; Others including Asian or Pacific Islander, and American Indian, Eskimo, or Aleut; reporting multiple races; whether the mother considered herself to be Hispanic or Latino; and unknown), based on maternal report of race and ethnicity at baseline
Mother marital status	Coded into six mutually exclusive indicators (single and never married; single and cohabitating; married; divorced or separated; other, including widowed; unknown), based on maternal report at baseline
Mother health	Dichotomous measure of whether mother's health is good or better, based on maternal report of health status as "excellent", "very good", or "good" as opposed to "fair" or "poor" at baseline
Mother depression	Sum of the Center for Epidemiologic Studies Depression Scale (CESD) items, based on maternal report at baseline
Cigarettes per week during pregnancy	Number of cigarettes smoked per week during pregnancy, based on maternal report at baseline
Alcohol drinks per week during pregnancy	Number of alcoholic drinks consumed per week during pregnancy, based on maternal report at baseline
Number of children born to mother	Number of children born to the same mother, based on maternal report at baseline
Number of adults in household	Number of adults in the household, based on maternal report at baseline
Biological father lives in household	Dichotomous measure of whether a biological father lives in the household, based on maternal report at baseline
Household combined income[b]	Combined household income from previous calendar year (see table note), coded into six mutually exclusive indicators (less than $10,000; $10,000 to $14,999; $15,000 to $19,999; $20,000 to $29,999; equal to or more than $30,000; and unknown), based on maternal report at baseline
Household net worth	Coded into six mutually exclusive indicators (debt is equal to or more than $5,000; debt is $0 to $4,999; breaking even; leftover is $0 to $4,999; leftover is equal to or more than $5,000; unknown), based on maternal report at baseline

© 2025 Duncan GJ et al. *JAMA Pediatr*.

Child age at interview	Child age in months at time of age 4 data collection. Age is calculated separately for age at time of maternal survey interview and age at time of BMI data collection
Site identifier	Dichotomous indicators of each of the four recruitment cities
Interviewer identifier	Dichotomous indicators of interviewer, based on a coded identifier for the individual interviewer who collected height and weight measurements (for BMI outcomes) or conducted the maternal survey (for survey outcomes) at age 4

[a] Covariates included in the primary model specification also include missing data indicators for the following baseline covariates: child weight at birth, child gestational age, cigarettes per week during pregnancy, and alcohol drinks per week during pregnancy.

[b] Household combined income is calculated to sum the total household income, government income (social program benefits such as Supplemental Security Income and Unemployment Insurance), and other income (business profit, aid from relatives, and child support benefits).

© 2025 Duncan GJ et al. *JAMA Pediatr*.

eTable 2. Descriptive Statistics and Cash Gift Impacts on Exploratory Maternal and Child Health Outcomes at Age 4

Panel A. Binary Outcomes[a]	Overall mean	Low-cash gift group mean	High-cash gift group mean[b]	Marginal effect (95% CI)[c]	*P* value	N
Maternal Mental Health						
**GAD-7**						
Meets Mild GAD-7 Threshold or Higher	0.410	0.390	0.438	0.057 (-0.005 to 0.119)	.07	883
Meets Moderate GAD-7 Threshold or Higher	0.174	0.157	0.198	0.046 (-0.005 to 0.096)	.08	883
Meets Severe GAD-7 Threshold	0.062	0.058	0.068	0.009 (-0.023 to 0.042)	.57	883
**PHQ-8**						
Meets Mild PHQ-8 Threshold or Higher	0.351	0.326	0.386	0.069 (0.008 to 0.130)	.03	883
Meets Moderate PHQ-8 Threshold or Higher	0.143	0.138	0.149	0.005 (-0.042 to 0.051)	.85	883
Meets Moderately Severe PHQ-8 Threshold or Higher	0.049	0.054	0.041	-0.021 (-0.051 to 0.010)	.18	883
Meets Severe PHQ-8 Threshold	0.012	0.017	0.005	-0.015 (-0.035 to 0.004)	.13	883
Maternal BMI						
Maternal BMI-Underweight	0.020	0.029	0.009	-0.026 (-0.059 to 0.007)	.12	737
Maternal BMI-Overweight	0.258	0.238	0.285	0.046 (-0.015 to 0.107)	.14	737
Maternal BMI-Obese	0.513	0.530	0.491	-0.041 (-0.113 to 0.031)	.26	737
Maternal BMI-Overweight or Obese	0.771	0.767	0.775	0.014 (-0.048 to 0.075)	.66	737
Child BMI Percentile						
Child BMI Percentile-Underweight	0.042	0.041	0.043	-0.001 (-0.030 to 0.028)	.96	811
Child BMI Percentile-Overweight	0.170	0.184	0.152	-0.022 (-0.073 to 0.029)	.40	811
Child BMI Percentile-Obese	0.192	0.184	0.203	0.015 (-0.038 to 0.068)	.57	811
Child BMI Percentile-Overweight or Obese	0.363	0.368	0.355	-0.005 (-0.072 to 0.062)	.88	811
Child Health Conditions						
Child Chronic Health Diagnosis: Any	0.077	0.068	0.089	0.021 (-0.015 to 0.058)	.24	884
Child Chronic Health Diagnosis: Asthma	0.035	0.029	0.043	0.010 (-0.016 to 0.037)	.44	884

Panel B. Continuous Outcomes[d]	Overall mean (SD)	Low-cash gift group mean (SD)	High-cash gift group mean (SD)[e]	Regression-adjusted difference in means (95% CI)[f]	*P* value	N
Child Overall Poor Health Index Items						
Maternal Evaluation of Child's Overall Poor Health	0.699 (0.899)	0.674 (0.888)	0.734 (0.915)	0.079 (-0.054 to 0.212)	.24	884
How Often Child is Sick	0.972 (0.835)	0.973 (0.843)	0.970 (0.825)	0.017 (-0.121 to 0.154)	.81	884

© 2025 Duncan GJ et al. *JAMA Pediatr*.

Abbreviations: SD, standard deviation; CI, confidence interval.
[a] In Panel A, each outcome is coded as a binary variable. See the text of eAppendices 2 and 4 for a discussion of the construction of each of these outcome measures.
[b] The proportion of respondents with each condition or meeting each clinical threshold is reported for the overall sample in column 1 and separately for the high-cash and low-cash gift groups in columns 2 and 3.
[c] Logistic regression models with site fixed effects and covariates are used to estimate cash gift impacts with the average marginal effect (or average marginal change in predicted probability of an affirmative response) reported in column 4 along with 95% confidence intervals constructed using robust standard errors. Because some of the outcomes are low probability events, some covariates perfectly predicted failure in the logistic regression models. For each outcome, covariates that perfectly predict failure are either combined with other covariates (for example, collapsing levels of education to fewer categories) or are dropped from the model. Interviewer IDs are dropped from all models. The exact lists of covariates used for each model are available upon request.
[d] In Panel B, each outcome variable is continuous and comprises two of the three measures included in the pre-registered additive index of child overall poor health (the third is child chronic health diagnosis, which is included in Panel A). See the text of eAppendices 2 and 4 for a discussion of the construction of each of these outcome measures.
[e] The mean for each outcome is reported for the overall sample in column 1 and separately for the high-cash and low-cash gift groups in columns 2 and 3 with standard deviations reported in parentheses.
[f] Ordinary Least Squares (OLS) regression models with site fixed effects and covariates are used to estimate cash gift impacts on each standardized outcome with effect sizes reported in column 3 along with 95% confidence intervals constructed using robust standard errors. Descriptions of each covariate used in the models can be found in eTable 1.

© 2025 Duncan GJ et al. *JAMA Pediatr*.

eTable 3. Cash Gift Impacts on Preregistered Maternal and Child Health Outcomes at Age 4 with Adjustments for Nonresponse[a]

	Unweighted (from Table 2)[b]		IPTW-ATT[c]		NRW-ATE[d]			Multiple Imputation (MI)[e]		
	Effect size (95% CI)[f]	*P* value[g]	Effect size (95% CI)	*P* value	Effect size (95% CI)	*P* value	Unweighted N[h]	Effect size (95% CI)	*P* value	N[i]
Preregistered Maternal Health Outcomes										
GAD-7 Total Score	0.12 (-0.02 to 0.25)	.09	0.08 (-0.07 to 0.23)	.29	0.10 (-0.03 to 0.24)	.14	883	0.11 (-0.02 to 0.25)	.10	995
PHQ-8 Total Score	0.04 (-0.08 to 0.17)	.51	-0.00 (-0.15 to 0.14)	.98	0.04 (-0.09 to 0.17)	.54	883	0.05 (-0.08 to 0.17)	.45	995
Mother's Body Mass Index	-0.06 (-0.21 to 0.09)	.42	-0.08 (-0.22 to 0.07)	.31	-0.04 (-0.19 to 0.11)	.59	737	-0.07 (-0.21 to 0.07)	.33	920
Preregistered Child Health Outcomes										
Child Overall Poor Health Index	0.08 (-0.07 to 0.22)	.30	0.10 (-0.04 to 0.25)	.16	0.07 (-0.07 to 0.21)	.32	884	0.07 (-0.07 to 0.20)	.34	995
Child's Body Mass Index Percentile	-0.03 (-0.17 to 0.12)	.73	0.05 (-0.11 to 0.20)	.57	-0.02 (-0.16 to 0.13)	.83	811	-0.01 (-0.15 to 0.14)	.90	995

Abbreviations: CI, confidence interval.

[a] All models were estimated using the same model specification as the main preregistered results presented in Table 2: Ordinary Least Squares (OLS) regressions with site-level fixed effects and covariates. For more details on the outcome measures, see eAppendix 2. For more details on the covariates included in the preregistered model specification, see eTable 1.

[b] Columns 1 and 2 come directly from columns 4 and 5 of Table 2.

[c] For the inverse probability of treatment weighted (IPTW) results presented in columns 3 and 4, low-cash gift group respondents were weighted by the inverse probability of being a high-cash gift group respondent to balance the baseline characteristics of low-cash gift group respondents to the characteristics of high-cash gift group respondents. The resulting estimand can be characterized as an "average treatment effect on the treated" or ATT.

[d] For the non-response weighted (NRW) results presented in columns 5 and 6, all respondents were weighted by the inverse probability of being an age 4 respondent to balance the baseline characteristics of respondents to the characteristics of the full BFY sample at baseline. The resulting estimand can be characterized as an "average treatment effect" or ATE. For more details on the construction of these weights, see the text of eAppendix 5.

[e] For the multiple imputation estimates, we imputed 20 datasets with multiple imputation by chained equations, or MICE, using predictive mean matching.

[f] Effect sizes (columns 1, 3, 5, and 8) were calculated by dividing the covariate-adjusted treatment effect by the standard deviation of the analysis sample low-cash gift group. Ninety-five percent confidence intervals are calculated using robust standard errors obtained from regression estimates.

[g] Unadjusted *P* values reported in columns 2, 4, 6 and 9 make no adjustments for multiple hypothesis testing.

[h] Unweighted Ns comes from the primary model specifications presented in Table 2.

[i] Ns for estimates using multiple imputation include all mothers and their children enrolled in the study at baseline with the exception of 5 cases in which the mother passed away during the study period and, for the maternal BMI outcomes, 75 mothers who were pregnant during age 4 data collection.

© 2025 Duncan GJ et al. *JAMA Pediatr*.

eTable 4. Sensitivity of Cash Gift Impacts on Maternal and Child Health Outcomes at Age 4 to Overpayments[a]

Panel A. Including overpayments as a covariate[b]	Low-cash gift group mean (SD)	High-cash gift group mean (SD)[c]	Regression-adjusted difference in means (95% CI)[d]	Standardized effect size (95% CI)[e]	*P* value	N
Pre-registered Maternal Health Outcomes						
GAD-7 Total Score	4.59 (4.91)	5.10 (5.02)	0.54 (-0.13 to 1.21)	0.11 (-0.03 to 0.25)	.11	883
PHQ-8 Total Score	4.21 (4.95)	4.46 (4.61)	0.19 (-0.44 to 0.82)	0.04 (-0.09 to 0.17)	.56	883
Mother's Body Mass Index	31.28 (8.43)	30.75 (7.83)	-0.51 (-1.74 to 0.72)	-0.06 (-0.21 to 0.09)	.41	737
Pre-registered Child Health Outcomes						
Child Overall Poor Health Index	1.71 (1.42)	1.79 (1.50)	0.10 (-0.10 to 0.30)	0.07 (-0.07 to 0.21)	.31	884
Child's Body Mass Index Percentile	64.64 (30.52)	63.85 (31.58)	-0.79 (-5.29 to 3.71)	-0.03 (-0.17 to 0.12)	.73	811
**Panel B. Dropping overpayment cases[f]**	**Low-cash gift group mean (SD)**	**High-cash gift group mean (SD)**	**Regression-adjusted difference in means (95% CI)[g]**	**Standardized effect size (95% CI)**	***P* value**	**N**
Pre-registered Maternal Health Outcomes						
GAD-7 Total Score	4.59 (4.93)	5.06 (5.01)	0.51 (-0.17 to 1.19)	0.10 (-0.03 to 0.24)	.14	861
PHQ-8 Total Score	4.21 (4.95)	4.44 (4.64)	0.18 (-0.46 to 0.82)	0.04 (-0.09 to 0.16)	.59	861
Mother's Body Mass Index	31.23 (8.44)	30.75 (7.80)	-0.48 (-1.72 to 0.76)	-0.06 (-0.20 to 0.09)	.45	722
Pre-registered Child Health Outcomes						
Child Overall Poor Health Index	1.72 (1.43)	1.77 (1.50)	0.08 (-0.12 to 0.28)	0.06 (-0.08 to 0.20)	.43	862
Child's Body Mass Index Percentile	64.78 (30.62)	63.82 (31.70)	-0.98 (-5.54 to 3.58)	-0.03 (-0.18 to 0.12)	.67	791

Abbreviations: SD, standard deviation; CI, confidence interval.
[a] Overpayments of the cash gift occurred for 22 families providing data in the age 4 sample (14 high-cash gift and 8 low-cash gift families). For more information on these overpayments, see the text of eAppendix 6. For more information on each outcome measure and its construction, see eAppendix 2.
[b] Panel A retains all observations in which an overpayment of the cash gift was ever received. For regression results, receipt of the cash gift overpayment is incorporated as a covariate in the models.
[c] The mean outcome of respondents is reported separately for the high-cash and low-cash gift groups in columns 1 and 2 with standard deviations reported in parentheses.
[d] Results in the third column come from OLS models that include site fixed effects, all pre-registered covariates included in the primary outcome specification in Table 2, as well as controls for receipt of an overpayment of the cash gift.
[e] Fourth column numbers convert third column differences into effect sizes with division by low-cash gift group standard deviations. Ninety-five percent confidence intervals are calculated using robust standard errors obtained from regression estimates.
[f] Results presented in Panel B drop all observations in which an overpayment of the cash gift was ever received, n=22 for the age 4 sample providing any data for health outcomes.
[g] Results in the third column come from OLS models that include site fixed effects as well as all pre-registered covariates included in the primary outcome specification in Table 2.

© 2025 Duncan GJ et al. *JAMA Pediatr*.
