# Supplementary material for: Cash Transfers and Their Effect on Maternal and Young Children’s Health: A Randomized Clinical Trial
Source: JAMA Pediatr. 2025 Jun 16;179(8):867–75. doi: 10.1001/jamapediatrics.2025.1612 (PMC12171960; doi:10.1001/jamapediatrics.2025.1612)
Supplement: Supplement 3. — Data Sharing Statement. [file jamapediatr-e251612-s003.pdf]

Data Sharing Statement

Duncan. Cash Transfers and Their Effect on Maternal and Young Children's Health. *JAMA Pediatr*. Published June 16, 2025. doi:10.1001/jamapediatrics.2025.1612

Data
Additional Information: Trial Registration: ClinicalTrials.gov: NCT03593356
Data available: Yes
Data types: Deidentified participant data, Data dictionary
How to access data: https://www.icpsr.umich.edu/web/DSDR/studies/37871/versions/V8
When available: With publication

Supporting Documents
Document types: Statistical/analytic code
How to access documents: Analytic code will be made available through openICPSR at the time of publication at the following URL:
https://www.openicpsr.org/openicpsr/project/159422/version/V4/view;jsessionid=4FE7D80A0F5918AD0BBE7E26BD8B00D7
When available: With publication

Additional Information
Who can access the data: Anyone requesting the data
Types of analyses: For any purpose
Mechanisms of data availability: Without investigator support
Any additional restrictions: Some data used in the article are not available owing to HIPPA restrictions
